# Knowledge of Alzheimer’s disease and associated factors among adults in Zhuhai, China: a cross-sectional analysis

**DOI:** 10.1186/s12889-024-19289-w

**Published:** 2024-07-03

**Authors:** Ya-Jun Sun, Jie Song, Xu-Ping Li, Xiao-Hui Wang, Yi-Xuan Wu, Jia-Ju Huang, Shi-Bin Wang, Yong-Yong Teng

**Affiliations:** 1 The Third People’s Hospital of Zhuhai, Zhuhai Center for Chronic Disease Prevention and Control, Zhuhai, Guangdong province 519000 China; 2 Henan International Collaborative Laboratory for Air Pollution Health Effects and Intervention, School of Public Health, Xinxiang Medical University, Xinxiang, 453003 China; 3 Xiangya School of Public Health, Central South University, No.172 Tongzipo Road, Yuelu District, Changsha City, Hunan Province 410006 China; 4 Guangdong Mental Health Center, Guangdong Provincial People’s Hospital (Guangdong Academy of Medical Sciences), Southern Medical University, Guangzhou, Guangdong province 510080 China

**Keywords:** Alzheimer’s disease, Public, Knowledge, Cross-sectional study

## Abstract

**Background:**

This study aimed to assess the public knowledge regarding Alzheimer’s Disease (AD) in Zhuhai, China, focusing on identifying knowledge gaps and the influence of demographic and health factors.

**Methods:**

A cross-sectional study was conducted in Zhuhai, China, from October to November 2022. A total of 1986 residents from 18 communities were selected employing stratified multi-stage equi-proportional sampling. Questionnaires covering general information and the Alzheimer’s Disease Knowledge Scale (ADKS) were investigated face-to-face. Ordinal multiclass logistic regression was applied to assess the relationship between AD awareness and demographic and health characteristics.

**Results:**

The average ADKS score was 18.5 (SD = 3.36) in Zhuhai. The lowest awareness rates were observed in the “Symptoms” and “Caregiving” subdomains of ADKS, with rates of 51.01% and 43.78%, respectively. The correct rates for the 30 ADKS questions ranged from 16.62 to 92.6%, showing a bimodal pattern with clusters around 80% and 20%. Women (OR = 1.203, 95% CI: 1.009–1.435), individuals aged 60 years or older (OR = 2.073, 95% CI: 1.467–2.932), those living in urban areas (OR = 1.361, 95% CI: 1.117–1.662), higher average monthly household income per capita (OR = 1.641, 95% CI: 1.297–2.082), and without any neurological or mental disorders (OR = 1.810, 95% CI: 1.323–2.478) were more likely to have higher levels of awareness about Alzheimer’s disease.

**Conclusions:**

Adults in Zhuhai show a limited knowledge of AD, particularly in the ‘Symptoms’ and ‘Caregiving’ subdomains. Upcoming health campaigns must focus on bridging the knowledge gaps in different subdomains of AD, especially among subgroups with lower awareness, as identified in our study.

**Supplementary Information:**

The online version contains supplementary material available at 10.1186/s12889-024-19289-w.

## Introduction

Alzheimer’s Disease (AD) is a progressively developing neurodegenerative disease characterized by memory loss, cognitive decline, and behavioral abnormalities [1]. AD is the most common cause of dementia, accounting for 50–75% of all dementia cases [2, 3]. The number of dementia patients was approximately 57.4 million in 2019, nearly doubling every 20 years, with most of the increase coming from developing countries [4]. China has the world’s largest number of dementia patients, and the number of dementia patients is also rapidly increasing [5]. According to a nationwide cross-sectional study in 2020, the prevalence of dementia among people aged 60 years and above in China was 6.0%, with approximately 65% suffering from AD [6]. In 2015, the total socio-economic cost of dementia in China was $167.74 billion, accounting for 1.47% of GDP, and is estimated to be $1.89 trillion by 2050 [7]. The significant challenge of preventing and treating AD in the country is exacerbated by the heavy burden of dementia and the public’s lack of awareness [8, 9].

In the latest research evidence [10–12], even in the most economically and medically advanced areas of eastern China, the level of knowledge about AD among medical personnel is also low. There are few people with a high level of knowledge, and a lack of knowledge about AD symptoms and nursing care. In terms of community healthcare workers, a survey from the central Chinese city of Changsha shows that despite their positive attitude towards the treatment of AD, there is still a lack of awareness about AD itself and its care modalities [13].

With the ongoing aging of China’s population, there has been growing attention from both government and society towards cognitive impairment such as AD. In 2020, the Chinese Office of the National Health Commission launched a strategic initiative to enhance specialized services for the prevention and treatment of AD [14]. This initiative sought to raise awareness about AD to 80% by 2022 [14]. Subsequently, in 2021, China’s major public health mental health project proposed strengthening public education and improving awareness about AD. However, targeted health education requires understanding the current public awareness level as a prerequisite [15].

Numerous studies have been conducted concerning AD awareness, primarily focusing on specific groups such as nursing and medical students, healthcare professionals, and nursing staff [11, 16–18]. However, research targeting the general adult population remains scarce. Two studies have reported the awareness level of AD prevention and treatment knowledge among Chinese residents over 18 years old by non-probability sampling method [19, 20]. However, these studies lack targeted guidance for conducting public health education on AD in Zhuhai due to differences in research populations, regional cultures, and sample representativeness.

The Alzheimer’s Disease Knowledge Scale (ADKS) can assess knowledge in a broad audience [21]. Garcia-Ribas et al. suggested that the independent items of the ADKS together constituted a comprehensive spectrum of information regarding AD knowledge [22]. Previous research illustrated the scale’s effectiveness and adaptability in a specific regional context within China [10, 13, 23]. This study aimed to use the probability sampling method to assess awareness and knowledge gaps regarding AD among adults in Zhuhai and investigate the influence of related factors on their awareness. The findings informed the development of targeted educational strategies and health promotion programs.

## Methods

### Study participants and sampling method

This survey was part of the 2022 Guangdong Province Resident Mental Health Literacy Survey Project conducted in Zhuhai from October to November 2022. The participants were residents of Zhuhai who were 18 years or older (born before September 1, 2004) and had lived in Zhuhai for at least six months within the past year.

**Fig. 1:**
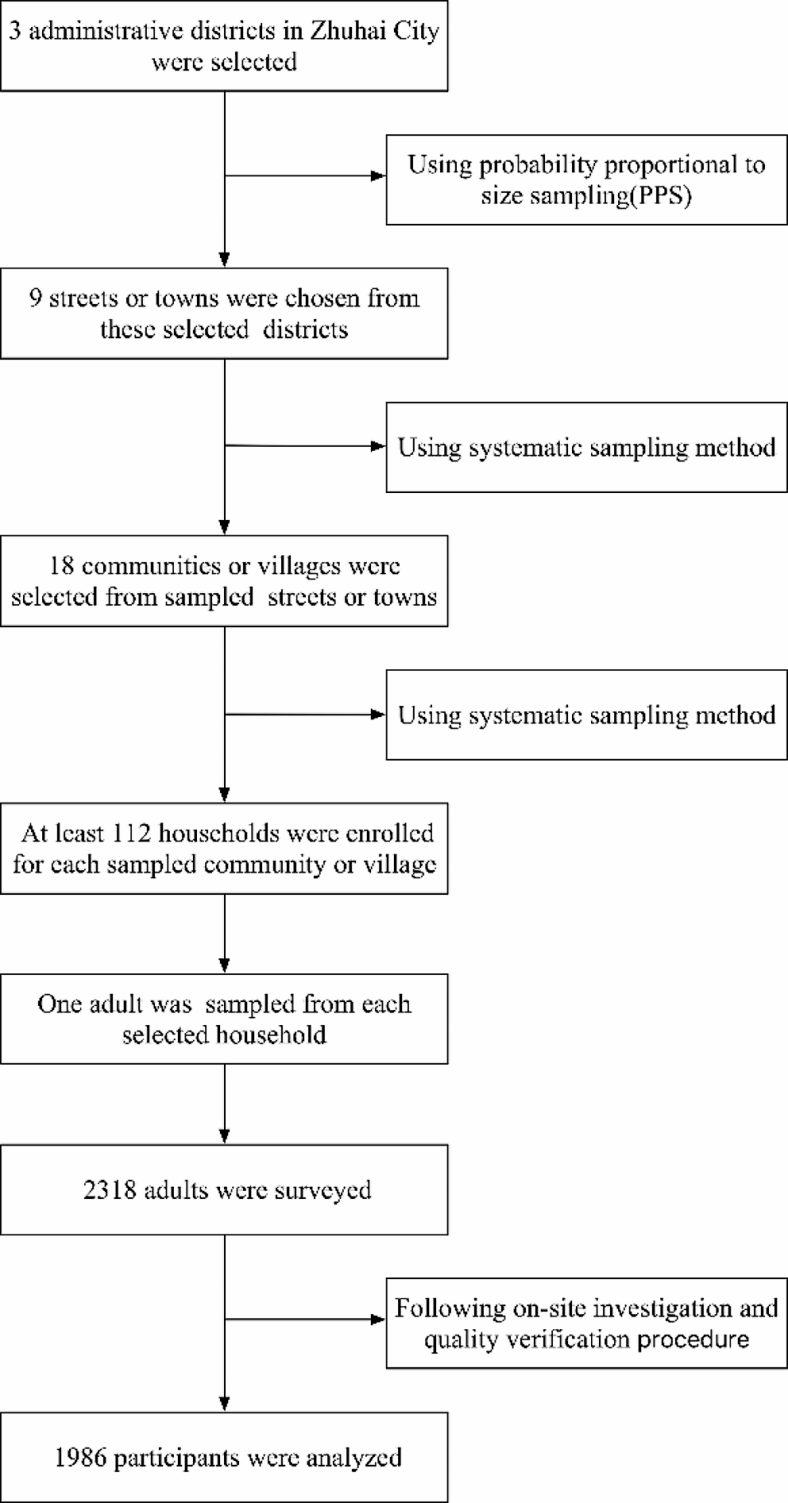
Flowchart of participants selection in this study

Participants were recruited utilizing a multi-stage stratified equal volume random sampling method, as illustrated in Fig. 1. Three administrative districts were identified as distinct strata within Zhuhai City. In the first stage, three streets or townships were randomly chosen from each administrative district based on population proportions. The second stage involved systematic sampling to select two communities or villages from each chosen street or township. In the third stage, 112 households were systematically sampled from each selected community or village, with one individual randomly chosen from each household for the survey.

If a household was ineligible or unreachable for various reasons, a replacement household with a similar family structure (gender, age, number of members) was selected from the same community or village. The closest one was chosen as a replacement if multiple similar households were available. The percentage of replaced samples was limited to 15%.

### Data collection and quality control

We recruited 18 psychiatric prevention doctors from community health service centers in nine streets or townships to serve as surveyors. Each surveyor was responsible for conducting surveys in one village or community. Participants completed the survey through face-to-face interviews using the Chinese online survey platform Questionnaire Star (URL: www.wjx.cn). The questionnaire collected data on participants’ demographic and health characteristics and AD knowledge. Demographic variables included age, gender, marital status, current residence, education level, monthly family income per capita, and history of neuropsychiatric diagnosis within the past year. AD knowledge was assessed using the Chinese ADKS, which consisted of 30 true/false questions across seven subdomains: symptoms, risk factors, course, assessment and diagnosis, caregiving, life impact, and treatment and management [21, 24]. Each correct answer received one point with a total score range of 0–30. The Cronbach’s alpha coefficients were 0.72 for the Chinese ADKS, which indicated good internal consistency reliability [25].

Several stringent quality control measures were implemented based on standardized training provided to the surveyors. Firstly, we used unique phone numbers to prevent multiple submissions from the same participant. Secondly, internal logic validation was applied to exclude responses that did not meet our criteria. Thirdly, a minimum threshold time for completing the questionnaire was set; any durations below this threshold were considered invalid responses. Lastly, a random subset of participants (5%) underwent re-interviews to verify the quality of their previous interviews.

### Statistical analysis

The analytical procedures were performed using R software (version 4.2.3). Quantitative variables were analyzed descriptively using mean, standard deviation (SD), median, and interquartile range. Qualitative variables were analyzed using frequency and percentage. Since the total ADKS score did not follow a normal distribution (Shapiro-Wilk test resulted in a p-value less than 0.05), it was transformed into an ordered categorical variable based on the interquartile range. Independence tests were conducted to assess the relationship between ADKS awareness level and each demographic variable, utilizing the “coin” package in the R language. Variables that yielded a p-value less than 0.05 were considered for subsequent multivariate analysis.

We categorized the ADKS score into three ordinal levels - low, medium, and high - based on the 60th and 80th percentiles to prevent information loss and ensure stability. Ordered multicategorical logistic regression, relying on the proportional odds assumption, enhances model precision and interpretability by leveraging the sequential nature of the dependent variable compared to standard logistic regression. The crucial proportional odds assumption, also known as the parallel regression assumption, posits that the impact of the explanatory variables remains consistent across all classification thresholds. When the sample size is very small within certain categories, or when there are few samples for certain combinations of explanatory variables, effectively testing this assumption can be challenging, and the model parameter estimation may become unstable.

We utilized an ordered multinomial logistic regression model from the MASS package to examine the relationship between ADKS awareness levels and various predictors and performed the Brant Omnibus test to validate this critical assumption (*p* > 0.05), ensuring the interpretability and accuracy of the model. The generalized variance inflation factor (GVIF) was calculated to assess multicollinearity among independent variables, indicating no severe multicollinearity with values below 10 [26, 27].

Participants with response durations exceeding one hour were excluded in the sensitivity analysis to evaluate model robustness. All statistical analyses were two-sided with a significance threshold of alpha = 0.05.

## Results

### Demographical characteristics of participants

Table 1 summarizes the participants’ characteristics. Initially, the survey included 2,318 individuals. Following on-site investigation and quality verification, the study retained 1,986 valid responses, yielding an 85.68% response rate. The respondents had an average age of 43.5 years (SD = 15.6) and a nearly equal male-to-female ratio (males: 49.9%, females: 50.1%). About 53.6% had a normal BMI range, and most were married, accounting for 74.8% of the sample population. Over 40% held at least a college or bachelor’s degree qualification, while around 6.75% reported being diagnosed with neurological or mental disorders in the past year. Approximately 42% had an average monthly household income per capita between CNY 3,500(US$ 487.21) and CNY 5,999(US$ 835.08).

**Table 1:** Descriptive statistics for characteristics of participants(*N* = 1986) †

Characteristic	*n*(%)	AD awareness levels *n*(%)			Z/χ^2^	*p*-value
		Low	Medium	High		
**Gender**					-1.985	0.047
Male	991 (49.9%)	552 (51.3%)	246 (50.8%)	193 (45.2%)		
Female	995 (50.1%)	523 (48.7%)	238 (49.2%)	234 (54.8%)		
**Age (years)**					23.789	< 0.001*
18–34	684 (34.4%)	354 (32.9%)	176 (36.4%)	154 (36.1%)		
35–44	489 (24.6%)	280 (26.0%)	126 (26.0%)	83 (19.4%)		
45–59	453 (22.8%)	269 (25.0%)	103 (21.3%)	81 (19.0%)		
≥ 60	360 (18.1%)	172 (16.0%)	79 (16.3%)	109 (25.5%)		
**Body mass index(BMI)**					0.451	0.798
< 18.5	149 (7.5%)	78 (7.3%)	38 (7.9%)	33 (7.7%)		
18.5–23.9	1065 (53.6%)	572 (53.2%)	280 (57.9%)	213 (49.9%)		
> 23.9	772 (38.9%)	425 (39.5%)	166 (34.3%)	181 (42.4%)		
**Residence**					-3.864	< 0.001*
Rural	579 (29.2%)	357 (33.2%)	117 (24.2%)	105 (24.6%)		
Urban	1,407 (70.8%)	718 (66.8%)	367 (75.8%)	322 (75.4%)		
**Educational level**					27.868	< 0.001*
Elementary School or Below	269 (13.5%)	137 (12.7%)	59 (12.2%)	73 (17.1%)		
Junior High School	385 (19.4%)	242 (22.5%)	82 (16.9%)	61 (14.3%)		
High School/ Vocational High School	500 (25.2%)	290 (27.0%)	119 (24.6%)	91 (21.3%)		
Associate/Bachelor’s Degree and Above	832 (41.9%)	406 (37.8%)	224 (46.3%)	202 (47.3%)		
**Marital status**	384 (19.3%)	190 (17.7%)	104 (21.5%)	90 (21.1%)	15.74	0.001*
Unmarried	1,486 (74.8%)	831 (77.3%)	359 (74.2%)	296 (69.3%)		
Married or living together	75 (3.8%)	31 (2.9%)	15 (3.1%)	29 (6.8%)		
Bereaved	41 (2.1%)	23 (2.1%)	6 (1.2%)	12 (2.8%)		
Divorce or separation	384 (19.3%)	190 (17.7%)	104 (21.5%)	90 (21.1%)		
**Average monthly family income per captia (CNY)**					14.215	0.003*
<=3499(US$ 487.07)	516 (26.0%)	324 (30.1%)	95 (19.6%)	97 (22.7%)		
3500–5999(US$ 487.21- US$ 835.08)	834 (42.0%)	430 (40.0%)	207 (42.8%)	197 (46.1%)		
6000–8999(US$ 835.22- US$ 1252.70)	387 (19.5%)	194 (18.0%)	118 (24.4%)	75 (17.6%)		
>=9000 (US$ 1252.83)	249 (12.5%)	127 (11.8%)	64 (13.2%)	58 (13.6%)		
**Neurological or mental disorders history in the past year**					-3.693	< 0.001*
Yes	134 (6.7%)	90 (8.4%)	31 (6.4%)	13 (3.0%)		
No	1,852 (93.3%)	985 (91.6%)	453 (93.6%)	414 (97.0%)		

†The ADKS score was categorized into three ordinal levels: “Low” (below the 60th percentile), “Medium” (60th to below 80th percentile), and “High” (80th percentile and above), due to its non-normal distribution (as indicated by the Shapiro-Wilk test)

*p-values are less than 0.05, based on *Chi-squared* or *Z* statistics

### ADKS scores and AD awareness rate

Table 2 shows the ADKS total and subdomain scores distribution for 1,986 participants. The average ADKS total score was 18.5 (SD = 3.36), with a mean correct response rate of 61.68%. Subdomain scores and response accuracies were as follows: Symptoms scored 2.04 (SD = 0.78) with an accuracy of 51.01% (second lowest); Risk Factors scored 3.92 (SD = 1.11) with an accuracy of 65.41%; Course scored 2.78 (SD = 0.87) with an accuracy of 69.54%; Assessment and Diagnosis scored 2.72 (SD = 0.71) with an accuracy of 68.06%; Caregiving scored 2.19 (SD = 1.10) with an accuracy of 43.78% (lowest); Life Impact scored 2.00 (SD = 0.80) with an accuracy of 66.65%; and Treatment and Management scored 2.85 (SD = 0.79) with an accuracy of 71.19% (highest).

**Fig. 2:**
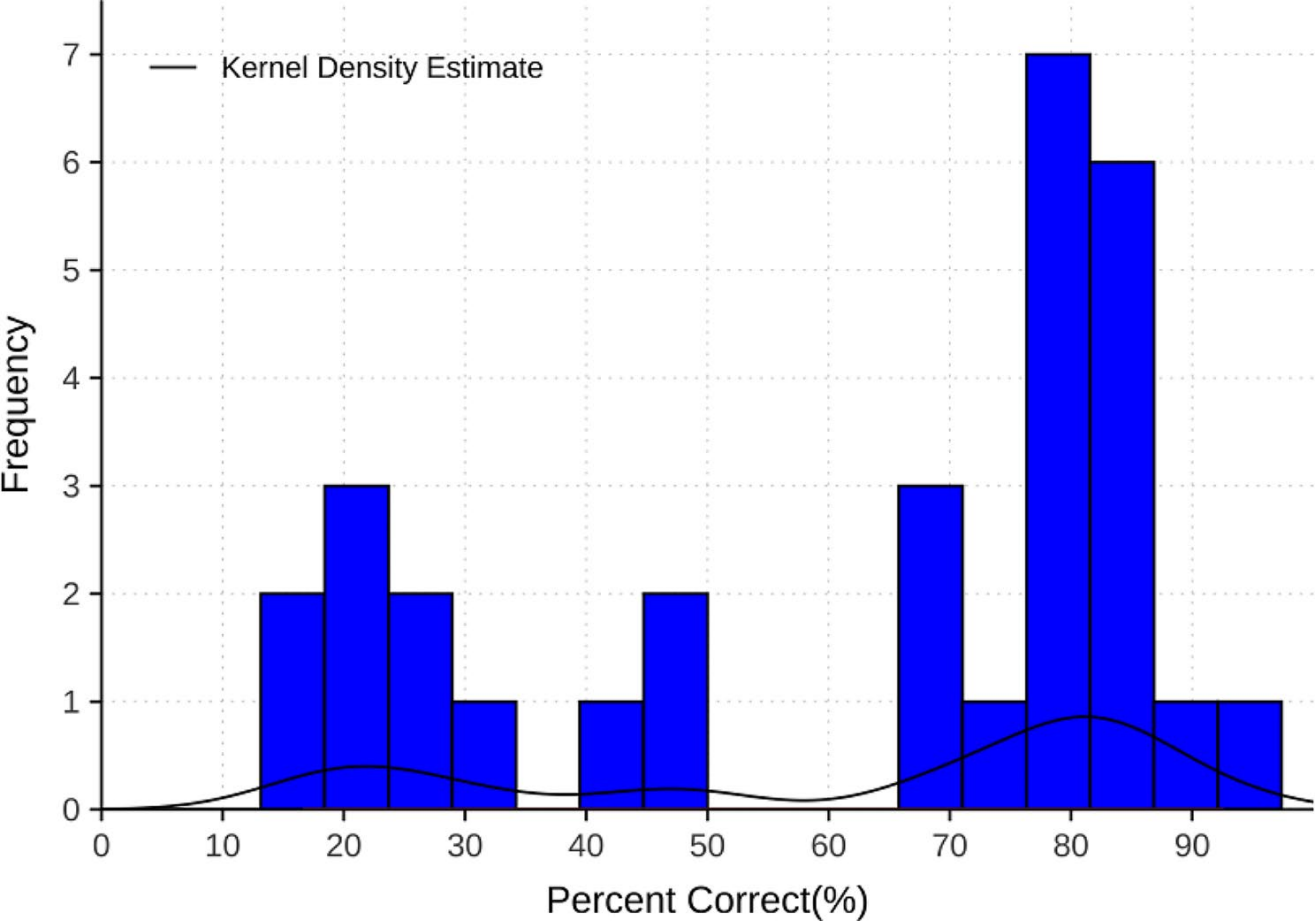
Distribution of correct answer percentages for ADKS individual questions

Table 3 presents the correct answer percentages for the 30 ADKS items, ranging from 16.62 to 92.6%. The top three items with high correct response rate were: “Alzheimer’s disease is one type of dementia” (92.60%), “A person with Alzheimer’s disease becomes increasingly likely to fall down as the disease gets worse” (91.49%), and “Poor nutrition can make the symptoms of Alzheimer’s disease worse” (85.40%). In contrast, the least understood items included: “If trouble with memory and confused thinking appears suddenly, it is likely due to Alzheimer’s disease” (16.62%), “It has been scientifically proven that mental exercise can prevent a person from getting Alzheimer’s disease” (17.57%), and “When people with Alzheimer’s disease repeat the same question or story several times, it is helpful to remind them that they are repeating themselves” (19.23%). The histogram in Fig. 2 shows a bimodal distribution of correct answer percentages, indicating two distinct clusters of questions: one cluster comprising around 12 (40%) questions with an average correct rate of about 80%, and another cluster consisting of 6 (20%) questions with an average accurate rate of approximately 20%. This bimodal pattern subtly reflects respondents’ perception regarding question difficulty or familiarity with the topics.

**Table 2:** Subdomain and total scores and average correct rates for ADKS

Content Domains	Score Range	Correct Answers (%)	Subdomain and Total Scores for ADKS						
			Mean (SD)	Min	P25	Median	P75	Max	
Treatment and Management	0–4	71.19	2.85 (0.79)	0	2	3	3	4	
Life Impact	0–3	66.65	2.00 (0.80)	0	1	2	3	3	
Course	0–4	69.54	2.78 (0.87)	0	2	3	3	4	
Assessment and Diagnosis	0–4	68.06	2.72 (0.71)	0	2	3	3	4	
Risk Factors	0–6	65.41	3.92 (1.11)	0	3	4	5	6	
Symptoms	0–4	51.01	2.04 (0.78)	0	2	2	2	4	
Caregiving	0–5	43.78	2.19 (1.10)	0	2	2	3	5	
Total	0–30	61.68	18.5 (3.36)	7	17	18	20	28	

**Table 3:** Alzheimer’s disease knowledge measured by the ADKS (by items, *n* = 1986)

Items	Number of correct answer (%)
1. People with Alzheimer’s disease are particularly prone to depression. (True)	1550 (78.05)
2. It has been scientifically proven that mental exercise can prevent a person from getting Alzheimer’s disease. (False)	349 (17.57)
3. After symptoms of Alzheimer’s disease appear, the average life expectancy is 6 to 12 years. (True)	1407 (70.85)
4. When a person with Alzheimer’s disease becomes agitated, a medical examination might reveal other health problems that caused the agitation. (True)	1682 (84.69)
5. People with Alzheimer’s disease do best with simple, instructions giving one step at a time. (True)	1554 (78.25)
6. When people with Alzheimer’s disease begin to have difficulty taking care of themselves, caregivers should take over right away. (False)	468 (23.56)
7. If a person with Alzheimer’s disease becomes alert and agitated at night, a good strategy is to try to make sure that the person gets plenty of physical activity during the day. (True)	1380 (69.49)
8. In rare cases, people have recovered from Alzheimer’s disease. (False)	646 (32.53)
9. People whose Alzheimer’s disease is not yet severe can benefit from psychotherapy for depression and anxiety. (True)	1655 (83.33)
10. If trouble with memory and confused thinking appears suddenly, it is likely due to Alzheimer’s disease. (False)	330 (16.62)
11. Most people with Alzheimer’s disease live in nursing homes. (False)	988 (49.75)
12. Poor nutrition can make the symptoms of Alzheimer’s disease worse. (True)	1696 (85.40)
13. People in their 30s can have Alzheimer’s disease. (True)	1600 (80.56)
14. A person with Alzheimer’s disease becomes increasingly likely to fall down as the disease gets worse. (True)	1817 (91.49)
15. When people with Alzheimer’s disease repeat the same question or story several times, it is helpful to remind them that they are repeating themselves. (False)	382 (19.23)
16. Once people have Alzheimer’s disease, they are no longer capable of making informed decisions about their own care. (False)	563 (28.35)
17. Eventually, a person with Alzheimer’s disease will need 24 h supervision. (True)	1654 (83.28)
18. Having high cholesterol may increase a person’s risk of developing Alzheimer’s disease. (True)	1680 (84.59)
19. Tremor or shaking of the hands or arms is a common symptom in people with Alzheimer’s disease. (False)	427 (21.50)
20. Symptoms of severe depression can be mistaken for symptoms of Alzheimer’s disease. (True)	1556 (78.35)
21. Alzheimer’s disease is one type of dementia. (True)	1839 (92.60)
22. Trouble handling money or paying bills is a common early symptom of Alzheimer’s disease. (True)	1541 (77.59)
23. One symptom that can occur with Alzheimer’s disease is believing that other people are stealing one’s things. (True)	1606 (80.87)
24. When a person has Alzheimer’s disease, using reminder notes is a crutch that can contribute to decline. (False)	945 (47.58)
25. Prescription drugs that prevent Alzheimer’s disease are available. (False)	883 (44.46)
26. Having high blood pressure may increase a person’s risk of developing Alzheimer’s disease. (True)	1662 (83.69)
27. Genes can only partially account for the development of Alzheimer’s disease. (True)	1620 (81.57)
28. It is safe for people with Alzheimer’s disease to drive, as long as they have a companion in the car at all times. (False)	1433 (72.16)
29. Alzheimer’s disease cannot be cured. (True)	1359 (68.43)
30. Most people with Alzheimer’s disease remember recent events better than things that happened in the past. (False)	478 (24.07)

### Awareness of Alzheimer’s disease and its association with demographic and health characteristics

Table 4 displays the relationship between levels of Alzheimer’s disease awareness and various demographic and health factors, as determined through multivariable ordered logistic regression analysis. Females (OR = 1.203, 95% CI: 1.009–1.435) showed a slightly higher likelihood of having increased AD awareness compared to males. Additionally, individuals aged 60 and above (OR = 2.073, 95% CI: 1.467–2.932) had significantly higher odds of being more aware of AD than those aged 18 to 34. Living in urban areas was linked to a higher level of AD awareness, with an odds ratio (OR) of 1.361 (95% CI: 1.117–1.662).

No significant difference was found in the odds of higher AD awareness among individuals with junior high education (OR = 0.776, 95% CI: 0.549–1.096), high school/vocational (OR = 0.963, 95% CI: 0.675–1.377), or associate/bachelor’s degrees and above (OR = 1.391, 95% CI: 0.958–2.024) compared to those with elementary education or lower levels. Likewise, the likelihood of possessing greater knowledge about AD was similar among married or cohabiting individuals (OR = 0.814, 95% CI: 0.630–1.051), bereaved individuals (OR = 1.403, 95% CI: 0.812–2.423), and divorced or separated individuals (OR = 1.112, 95% CI: 0.568–2.128) when contrasted with unmarried persons.

Moreover, a higher monthly household income per capita was associated with increased AD awareness; participants with a monthly household income per capita between CNY 3,500 - CNY 5,999 (OR = 1.641, 95% CI: 1.297–2.082) had higher chances for elevated awareness about AD compared to those earning less than CNY 3499. However, this trend did not continue for participants with even higher incomes above CNY 5,999.

Furthermore, individuals without a history of neurological or mental disorders in the past year were significantly more likely to have heightened Alzheimer’s Disease awareness compared to those with such a history, showing an OR value of 1.931 (95% CI: 1.345–2.820).

Multicollinearity among demographic and health variables was assessed during the analysis; results from Supplementary Table 1 indicate minimal concerns regarding multicollinearity, as all GVIF values are well below the commonly accepted threshold.

### Sensitivity analysis

To evaluate the robustness of the results, a sensitivity analysis was conducted by excluding participants who took one hour or longer to respond [28]. Primary predictive variables retained their original significance and directionality concerning AD awareness level after excluding these participants. In-depth outcomes can be found in Supplementary Table 2.

**Table 4:** Ordered logistic regression analysis on the association of demographic and health factors with AD knowledge levels†

Characteristic	B	SE	Wald χ^2^	OR(95%CI)	*P*
**Gender (ref: Male)**					
Female	0.185	0.090	4.245	1.203 (1.009–1.435)	0.039*
**Age (years) (ref: 18–34)**					
35–44	-0.099	0.128	0.597	0.906 (0.704–1.164)	0.440
45–59	0.077	0.143	0.292	1.080 (0.816–1.431)	0.589
≥ 60	0.729	0.177	17.052	2.073 (1.467–2.932)	< 0.001*
**Residence (ref: Rural)**					
Urban	0.309	0.101	9.275	1.361 (1.117–1.662)	0.002*
**Educational level (ref: Elementary School or Below)**					
Junior High School	-0.254	0.176	2.077	0.776 (0.549–1.096)	0.150
High School/ Vocational High School	-0.037	0.182	0.043	0.963 (0.675–1.377)	0.837
Associate/Bachelor’s Degree and Above	0.330	0.191	2.996	1.391 (0.958–2.024)	0.083
**Marital status (ref: Unmarried)**					
Married or living together	-0.206	0.130	2.501	0.814 (0.630–1.051)	0.114
Bereaved	0.339	0.279	1.479	1.403 (0.812–2.423)	0.224
Divorce or separation	0.106	0.335	0.100	1.112 (0.568–2.128)	0.752
**Average monthly family income per captia (CNY)** **(ref:<=3499(US$ 487.07))**					
3500–5999(US$ 487.21- US$ 835.08)	0.495	0.121	16.851	1.641 (1.297–2.082)	< 0.001*
6000–8999(US$ 835.22- US$ 1252.70)	0.506	0.144	12.396	1.659 (1.252–2.201)	< 0.001*
>=9000 (US$ 1252.83)	0.486	0.165	8.622	1.625 (1.175–2.247)	0.003*
**Neurological or mental disorders history in the past year** **(ref: Yes)**					
No	0.658	0.188	12.195	1.931 (1.345–2.820)	< 0.001*

† *B*: Ordinal logistic regression model βeta-estimates; *SE*: Standard Errors; *Wald χ*^*2*^: Wald Chi-Squared Test; *OR (95%CI)*: Ordinal logistic regression Odds Ratios with 95% Confidence Intervals; *P*: p-values, an asterisk (*) indicates < 0.05

## Discussion

Our study investigated public awareness of Alzheimer’s Disease (AD) and identified significant knowledge gaps and their causes. Participants showed a low overall level of AD awareness, with an average ADKS score of 18.50, representing an awareness rate of 61.68%. Among the seven subdomains of ADKS, “symptoms” and “caregiving” had the lowest awareness rates. Individual question accuracy rates displayed a bimodal pattern distribution. Furthermore, the level of awareness about AD was associated with different demographic and health factors.

Several existing studies using ADKS to assess the general population’s knowledge of AD produced inconsistent results across different countries [29–32]. In our research involving Zhuhai’s adult population, the average ADKS score was 18.5 (SD = 3.36), aligning with previous studies in Spain and Lebanon [29, 30] but notably lower than Brazil’s average score of 21.6 (SD = 3.73) due to the inclusion of healthcare professionals who typically have higher AD awareness [31], and surpassing Saudi Arabia’s average of 17.35 (SD = 3.1), as reported by Alorfi, likely due to not including participants over age sixty [32].

In this study, the correct response rates for individual items on the ADKS exhibited a clear bimodal pattern distribution, indicating substantial gaps in participants’ understanding regarding different aspects of Alzheimer’s disease [33, 34]. Within the seven subdomains of ADKS, participants demonstrated relatively lower awareness in “Symptoms” and “Caregiving,” consistent with observations made by Ma et al. among community health service center staff in Jiaxing, China [10]. A possible reason for this knowledge gap could be that public education initiatives, media coverage, and healthcare system educational programs may have disproportionately emphasized other aspects while neglecting “Symptoms” and “Caregiving.” Consequently, this leads to disparities in public awareness levels concerning these areas due to an imbalanced information environment.

In this study, gender, age, place of residence, and monthly household income per capita were significantly associated with AD awareness. Women and participants aged 60 and older exhibited higher levels of awareness in line with prior research findings [29, 34–37]. This could be due to women’s traditional caregiving roles, their tendency to seek health information [38–40], sociocultural influences [38], as well as older individuals’ increased exposure to AD, targeted awareness campaigns, and accumulated health knowledge over time [41]. Although women had a slight but statistically significant advantage in AD knowledge, the differences suggest minimal practical implications based on gender. Our findings indicate urban residents had higher knowledge levels than their rural counterparts, consistent with a 2019 study in China [19]. However, this result contrasts with several international studies that found no significant link between residence and knowledge levels according to the ADKS [18, 29, 42]. The discrepancy between Chinese and international studies can be attributed to China’s distinct socio-economic landscape, particularly in urban areas where residents have better access to healthcare and increased exposure to health education campaigns, potentially enhancing their disease awareness like AD [43]. Additionally, participants with a monthly household income per capita of at least CNY 3500 (approximately US$ 487.21) showed greater AD awareness compared to those earning less, aligning with prior research by Elbejjani et al. and Werner [30, 41]. This trend may be due to higher-income individuals being more proactive in seeking health-related information [44].

This study did not find a significant link between educational attainment and AD awareness. This finding contrasts with earlier studies, which typically indicate that higher education levels are associated with greater AD knowledge [30, 32, 45]. Although individuals with higher education might have better comprehension skills for understanding complex health issues, our data suggest that education level does not directly impact AD awareness [46]. Similarly, our research revealed no notable link between marital status and levels of AD knowledge, consistent with previous studies [47–49]. In contrast, a study conducted in Northern Ireland found a significant association between marital status and AD knowledge levels, potentially influenced by the age demographics of the participants [36]. Another study in Iran indicated that marital status substantially impacts health literacy, encompassing health knowledge, with married individuals exhibiting more excellent proficiency [48]. The disparities in these findings may arise from differences in methodology, demographics, and cultural attitudes toward marriage [48].

Our study made an intriguing observation: a negative association exists between a diagnosis of neurological or mental disorders in the past year and the awareness level as measured by the ADKS. Specifically, participants diagnosed with these conditions in the past year showed significantly lower understanding levels than those without such a diagnosis. This result indicates our study has uncovered a novel area deserving further investigation. A plausible explanation is that individuals with neurological or mental disorders may face barriers to accessing reliable information or understanding complex medical terms due to cognitive limitations [50]. Furthermore, the stigma surrounding these disorders could additionally impede their quest for knowledge about Alzheimer’s Disease [50]. Given this finding, it is prudent to give special attention to individuals with a history of neurological or mental disorders when sharing information about Alzheimer’s Disease, to prevent further marginalization.

The study has several advantages. The sample of this study had higher representativeness because it adopted a strict stratified cluster random sampling method and face-to-face interviews, resulting in a higher response rate. Additionally, on-site instant communication and clarification contributed to a better understanding and quality of questionnaire completion, thereby enhancing the accuracy of research results.

The present study also has several limitations that need to be considered. Firstly, using non-anonymous surveys may lead to a social desirability bias where participants provide more positive responses, inaccurately reporting a history of neurological or mental disorders due to the stigma associated with such conditions. Secondly, factors like relatives with AD, medical resource access, health information reception, cultural beliefs, and cognitive engagement significantly impact levels of AD knowledge, along with sociodemographic characteristics, but these were not accounted for in the questionnaire design [51–53]. Thirdly, despite utilizing a rigorous multi-stage stratified equal volume random sampling method, substituting participants during on-site investigations could have introduced bias. Moreover, there may be recall bias in data collection due to participants potentially inaccurately recalling past health factors and knowledge. Fourthly, the cross-sectional nature of this study means causal relationships can not be inferred; it only captures associations between variables at one specific time. Lastly, surveying one city limits its generalizability across regions with varying public health resources and awareness campaigns. Despite these limitations, the study provides valuable insights and suggests directions for future research. Future studies could: (1) Conduct qualitative research to deepen understanding of factors leading to low awareness in areas such as “Symptoms” and “Caregiving,” guiding tailored health education strategies; (2) Investigate specific populations with limited awareness (e.g., low-income individuals, and those with neurological or mental disorders) to uncover unique challenges and propose effective solutions.

## Conclusions

In Zhuhai, adults showed limited knowledge of AD, particularly regarding its symptoms and caregiving. Awareness varied significantly across different demographic groups. These findings are significant for policymakers and healthcare providers in the region. Launching targeted health promotion campaigns is recommended to bridge these knowledge gaps, especially focusing on the subgroups with lower awareness levels identified in this study. These initiatives should educate the public on the early signs and symptoms of AD and inform them about available caregiving resources and support services. Furthermore, the results highlight the need for healthcare providers to customize their education and outreach to reach and engage communities with lower AD awareness levels effectively.

### Electronic supplementary material

Below is the link to the electronic supplementary material.

Supplementary Material 1

Supplementary Material 2

## Data Availability

The datasets generated and/or analyzed during the current study are not publicly available due to privacy and confidentiality concerns regarding sensitive personal information, but are available from the corresponding author on reasonable request.
