# Supplementary material for: Knowledge of Alzheimer’s disease and associated factors among adults in Zhuhai, China: a cross-sectional analysis
Source: BMC Public Health. 2024 Jul 3;24:1769. doi: 10.1186/s12889-024-19289-w (PMC11220978; doi:10.1186/s12889-024-19289-w)
Supplement: Supplementary file 1 — Supplementary Material 1 [file 12889_2024_19289_MOESM1_ESM.doc]

Supplementary Table 1: The Multicollinearity Test Results

| **Variables** | **GVIF** | **Df** | **GVIF^(1/(2*Df))^** |
| --- | --- | --- | --- |
| **Gender** | 1.058 | 1 | 1.029 |
| **Age** | 2.561 | 3 | 1.170 |
| **Residence** | 1.051 | 1 | 1.025 |
| **Educational level** | 2.281 | 3 | 1.147 |
| **Marital status** | 1.693 | 3 | 1.092 |
| **Average monthly family income per capita** | 1.331 | 3 | 1.049 |
| **Neurological and mental disorders history in the past year** | 1.021 | 1 | 1.010 |
