# Supplementary material for: Knowledge of Alzheimer’s disease and associated factors among adults in Zhuhai, China: a cross-sectional analysis
Source: BMC Public Health. 2024 Jul 3;24:1769. doi: 10.1186/s12889-024-19289-w (PMC11220978; doi:10.1186/s12889-024-19289-w)
Supplement: Supplementary file 2 — Supplementary Material 2 [file 12889_2024_19289_MOESM2_ESM.doc]

Supplementary Table 2: The sensitivity analysis for the association between predictor variables and Alzheimer's disease knowledge levels†

| **Characteristic** | ***B*** | ***SE*** | ***Wald χ^2^*** | ***OR*(95%*CI*)** | ***P*** |
| --- | --- | --- | --- | --- | --- |
| **Gender (ref: Male)** |  |  |  |  |  |
| Female | 0.183 | 0.091 | 4.045 | 1.201 (1.005-1.435) | 0.044* |
| **Age** **(years) (ref:** **18-34)** |  |  |  |  |  |
| 35-44 | -0.085 | 0.129 | 0.437 | 0.918 (0.713-1.183) | 0.509 |
| 45-59 | 0.068 | 0.145 | 0.221 | 1.070 (0.806-1.422) | 0.639 |
| ≥60 | 0.759 | 0.178 | 18.262 | 2.136 (1.509-3.028) | <0.001* |
| **Residence (ref: Rural)** |  |  |  |  |  |
| Urban | 0.300 | 0.102 | 8.594 | 1.349 (1.105-1.650) | 0.003* |
| **Educational level (ref: Elementary school or below)** |  |  |  |  |  |
| Junior High School | -0.257 | 0.178 | 2.091 | 0.773 (0.546-1.096) | 0.148 |
| High School/ Vocational High School | -0.060 | 0.184 | 0.107 | 0.941 (0.657-1.352) | 0.743 |
| Associate/Bachelor's Degree and Above | 0.314 | 0.192 | 2.665 | 1.369 (0.940-1.999) | 0.103 |
| **Marital status (ref: Unmarried)** |  |  |  |  |  |
| Married or living together | -0.204 | 0.132 | 2.386 | 0.816 (0.630-1.057) | 0.122 |
| Bereaved | 0.345 | 0.280 | 1.517 | 1.412 (0.814-2.447) | 0.218 |
| Divorce or separation | 0.154 | 0.338 | 0.208 | 1.166 (0.594-2.246) | 0.649 |
| **Average monthly family income per capita (CNY)**  **(ref:<=3499(US$487.07))** |  |  |  |  |  |
| 3500-5999(US$487.21- US$835.08) | 0.497 | 0.122 | 16.494 | 1.644 (1.295-2.093) | <0.001* |
| 6000-8999(US$835.22- US$1252.70) | 0.520 | 0.146 | 12.623 | 1.681 (1.263-2.241) | <0.001* |
| >=9000 (US$1252.83) | 0.493 | 0.167 | 8.682 | 1.638 (1.179-2.274) | 0.003* |
| **Neurological and mental disorders history in the past year**  **(ref:Yes)** |  |  |  |  |  |
| No | 0.658 | 0.193 | 11.636 | 1.932 (1.333-2.847) | 0.001* |

† *B*: Ordinal logistic regression model βeta-estimates; *SE*: Standard Errors; *Wald χ^2^*: Wald Chi-Squared Test; *OR (95%CI)*: Ordinal logistic regression Odds Ratios with 95% Confidence Intervals; *P*: p-values, an asterisk (*) indicates <0.05.
